# Multi-hole metal stent can prevent cystic and pancreatic duct obstruction during endoscopic ultrasound-guided antegrade stenting combined with hepaticogastrostomy

**DOI:** 10.1055/a-2528-0340

**Published:** 2025-02-17

**Authors:** Takeshi Ogura, Yuki Uba, Takafumi Kanadani, Kimi Bessho, Hiroki Nishikawa

**Affiliations:** 1 Endoscopy Center, Osaka Medical and Pharmaceutical University Hospital, Osaka, Japan; 2 2nd Department of Internal Medicine, Osaka Medical and Pharmaceutical University, Osaka, Japan

Endoscopic ultrasound-guided biliary drainage (EUS-BD) is mainly indicated as an alternative to endoscopic retrograde cholangiopancreatography (ERCP) in patients with an inaccessible papilla. Recently, because of improved systemic chemotherapy including immune checkpoint inhibitors, a longer duration of stent patency has become very important even in EUS-BD. EUS-guided antegrade stenting (EUS-AS) combined with hepaticogastrostomy using a self-expandable metal stent (SEMS) can achieve longer-duration stent patency 1 2 . In EUS-AS, to prevent stent dislocation, cholecystitis, and pancreatitis, an uncovered SEMS is usually selected. However, an uncovered SEMS has several disadvantages, such as easy tumor ingrowth, which could shorten its patency in comparison with a fully covered SEMS (FCSEMS). If, on the other hand, a fully covered SEMS is deployed antegradely, cystic duct or pancreatic duct obstruction can occur. Recently, a fully covered SEMS with side holes (Hanarostent Biliary Multi-hole NEO; M. I. Tech Co., Ltd., Pyeongtaek, South Korea) has become available (MHSEMS) ( Fig. 1 ). This stent was designed to prevent stent migration by means of the small tissue ingrowths that form in the multiple small (1.8-mm) side holes along the covering membrane 3 4 . Through this design, a MHSEMS can prevent the adverse events mentioned. Technical tips for EUS-AS combined with hepaticogastrostomy using a MHSEMS are presented here.

**Fig. 1:**
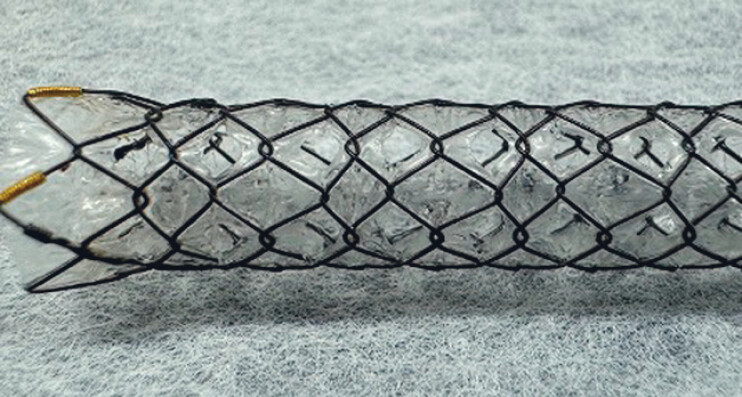
A fully covered self-expandable metal stent with side holes.

A 73-year-old man was admitted to our hospital due to obstructive jaundice caused by bile duct cancer. Because of malignant duodenal obstruction, EUS-BD was attempted. First, the intrahepatic bile duct was punctured using a 19-G needle, and contrast medium was injected. A 0.025-inch guidewire was inserted into the biliary tract, and an ERCP catheter was inserted. After contrast medium injection, middle common bile duct obstruction was observed ( Fig. 2 ). In addition, the cystic duct was observed near the stricture site ( Fig. 3 ). After guidewire placement within the intestine, a MHSEMS was deployed from the intestine to the upper common bile duct ( Fig. 4 ). Finally, a partially covered SEMS was deployed from the intrahepatic bile duct to the stomach. No adverse events occurred during the procedure ( Fig. 5 ; Video 1 ). Neither stent obstruction, acute cholecystitis, nor pancreatitis was observed during 6-month follow-up.

**Fig. 2:**
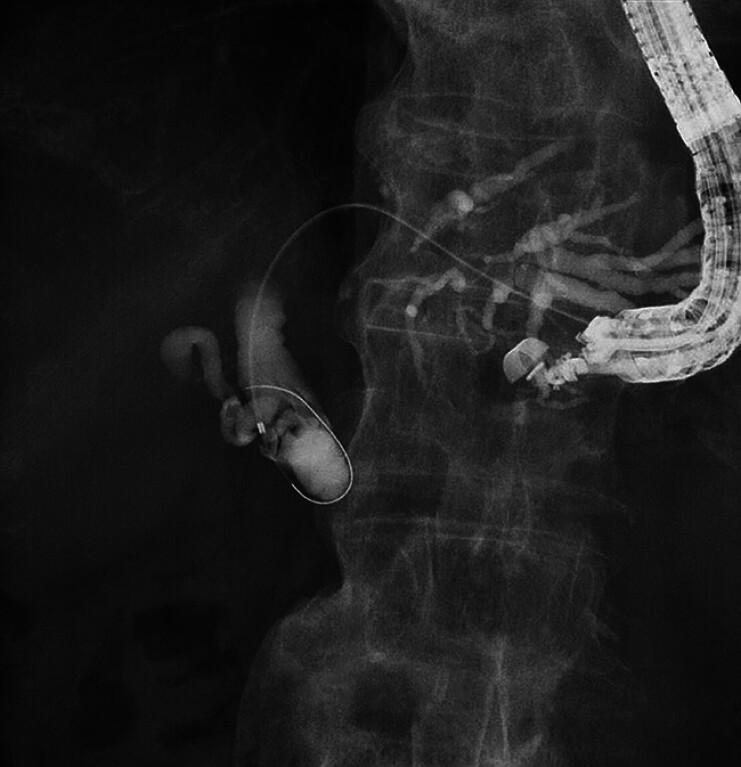
Endoscopic retrograde cholangiopancreatography. After contrast medium injection, middle common bile duct obstruction is observed.

**Fig. 3:**
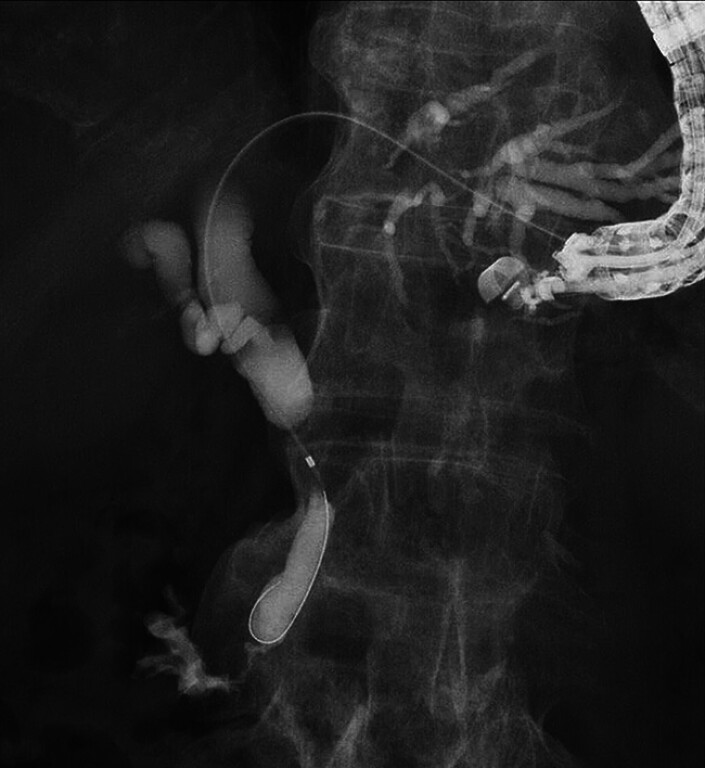
The cystic duct is observed near the stricture site.

**Fig. 4:**
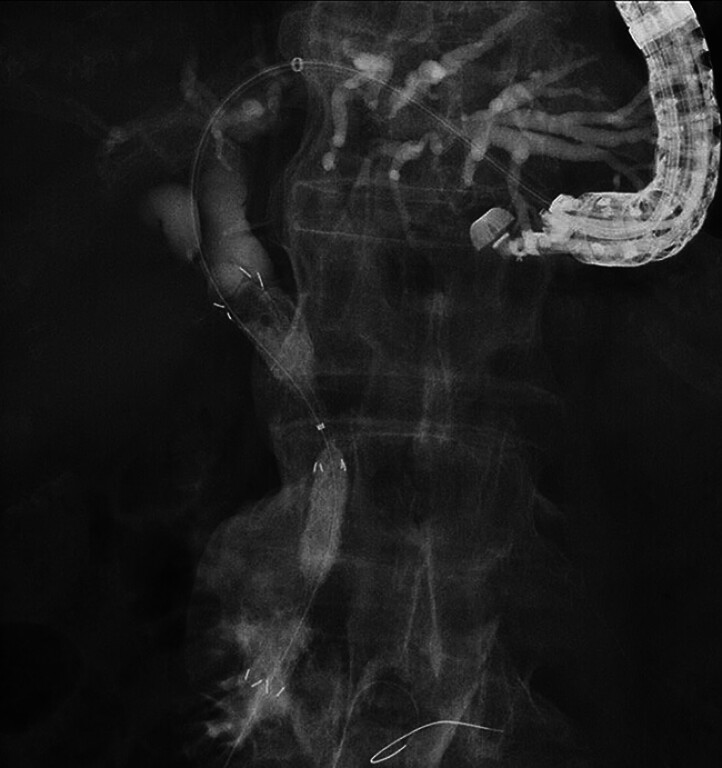
After guidewire placement within the intestine, a multi-hole self-expandable metal stent is deployed from the intestine to the upper common bile duct.

**Fig. 5:**
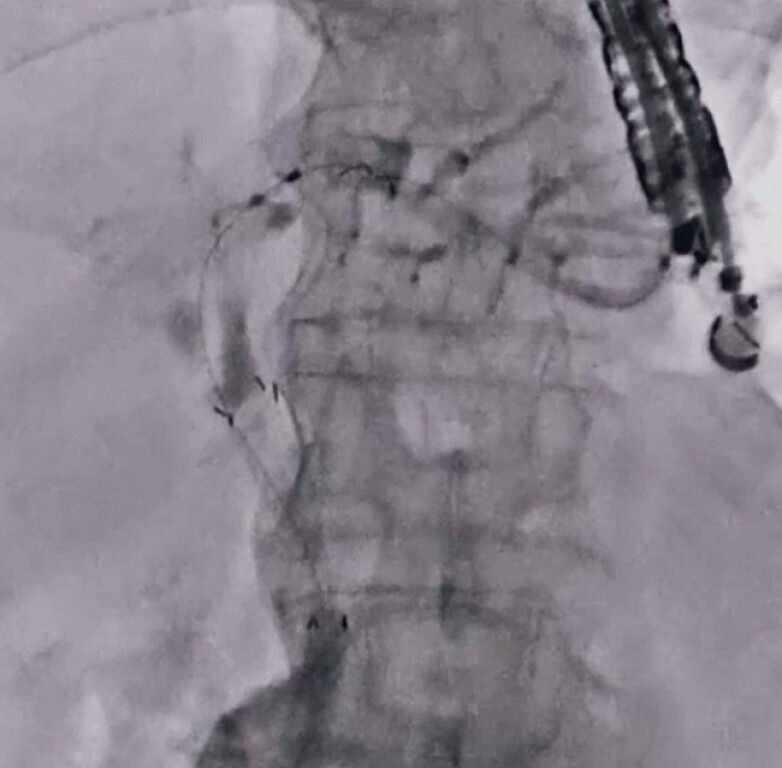
A partially covered self-expandable metal stent is deployed from the intrahepatic bile duct to the stomach.

A multi-hole self-expandable metal stent deployed from the intestine to the upper common bile duct can prevent cystic and pancreatic duct obstruction during endoscopic ultrasound-guided antegrade stenting combined with hepaticogastrostomy.Video 1

In conclusion, a MHSEMS may prevent cystic duct or pancreatic duct obstruction during EUS-AS, although further reports are needed to verify the usefulness of the MHSEMS.

Endoscopy_UCTN_Code_TTT_1AR_2AZ

## Notes

Endoscopy E-Videos https://eref.thieme.de/e-videos
*E-Videos* is an open access online section of the journal *Endoscopy* , reporting on interesting cases and new techniques in gastroenterological endoscopy. All papers include a high-quality video and are published with a Creative Commons CC-BY license. Endoscopy E-Videos qualify for HINARI discounts and waivers and eligibility is automatically checked during the submission process. We grant 100% waivers to articles whose corresponding authors are based in Group A countries and 50% waivers to those who are based in Group B countries as classified by Research4Life (see: https://www.research4life.org/access/eligibility/ ). This section has its own submission website at https://mc.manuscriptcentral.com/e-videos .
